# Genomic Sequences of two Novel *Levivirus* Single-Stranded RNA Coliphages (Family *Leviviridae*): Evidence for Recombinationin Environmental Strains

**DOI:** 10.3390/v4091548

**Published:** 2012-09-13

**Authors:** Stephanie D. Friedman, Wyatt C. Snellgrove, Fred J. Genthner

**Affiliations:** 1 US Environmental Protection Agency, Gulf Ecology Division, 1 Sabine Island Drive, Gulf Breeze, FL, 32561, USA; Email: friedman.stephanie@epa.gov (S.D.F.); genthner.fred@epa.gov (F.J.G.); 2 William Carey University College of Osteopathic Medicine, 498 Tuscan Avenue, Hattiesburg, MS 39401, USA; Email: cliffsnellgrove@gmail.com

**Keywords:** male-specific coliphage, *Leviviridae*, viral recombinants, ssRNA virus, FRNA, bacteriophage

## Abstract

Bacteriophages are likely the most abundant entities in the aquatic environment, yet knowledge of their ecology is limited. During a fecal source-tracking study, two genetically novel *Leviviridae* strains were discovered. Although the novel strains were isolated from coastal waters 1130 km apart (North Carolina and Rhode Island, USA), these strains shared 97% nucleotide similarity and 97–100% amino acid similarity. When the novel strains were compared to nine *Levivirus* genogroup I strains, they shared 95–100% similarity among the maturation, capsid and lysis proteins, but only 84–85% in the RNA-dependent RNA polymerase gene. Further bioinformatic analyses suggested a recombination event occurred. To the best of our knowledge, this is the first description of viral recombinants in environmental *Leviviridae* ssRNA bacteriophages.

## 1. Introduction

Bacteriophages have played a major role contributing to our knowledge of molecular biology, not only in the role of model viruses but also as tools to investigate mRNA, genes, genetic codes and genomes. The first sequenced genomes were the RNA bacteriophage MS2 [1] and the DNA bacteriophage Ф-X174 [2]. As important as *Drosophila* was in shaping the field of genetics and Tobacco Mosaic Virus was in advancing the study of virology and biochemistry, the RNA phage MS2 (family *Leviviridae*) was fundamental in laying the foundation of molecular biology. Thus, it is important to continue adding to the basic understanding of phages. The observations presented in this study were rather serendipitous, in that the focus was not on searching for natural recombinant bacteriophages. Nonetheless, evidence of a recombination event was revealed during a ssRNA bacteriophage sequencing project [3]. 

Male-specific ssRNA (FRNA) coliphages belong to the family *Leviviridae*. They are classified into two genera (*Levivirus* and *Allolevivirus*) which are subdivided into four genogroups (genogroups I and II in *Levivirus* and genogroups III and IV in *Allolevivirus*). Investigating the genetic diversity of FRNA phages Vinjé *et al.* [4] conducted a phylogenetic analysis of 32 *Levivirus* field strains using a 189 bp replicase gene fragment. This study revealed three main clusters: genogroup I, genogroup II and a potential novel group, designated JS, which clustered between genogroup I and genogroup II. The putative JS group, represented by phages, WWTP1_50 and 2GI13, had a >40% sequence diversity in the 189 bp replicase gene sequence when compared to strains from genogroups I and II. As these strains were isolated from widely separated geographical regions (Massachusetts and South Carolina) Vinjé *et al*., [4] proposed that JS may form a stable lineage. This report suggested further genomic sequencing and serological data were needed to confirm whether these strains formed a novel genogroup or whether they were the result of recombination or rearrangement events [5]. 

In its simplest form, recombination occurs when two disparate DNA or RNA strands exchange or merge stretches of their sequences whereas mutation involves the substitution, deletion or insertion of a nucleotide resulting in the change of the nucleotide sequence of a gene or an amino acid sequence of a protein. In some RNA viruses, RNA recombination events can occur when two or more strains infect the same host. Proposed models for the formation of novel RNA sequences include (i) cleavage and ligation in RNA molecules or RNA secondary structures [6], (ii) replicative template switching whereby the RNA-dependent RNA polymerase (replicase) switches from one template to another RNA template, also known as copy choice [5,7], and (iii) RNA transesterification which occurs when the polymerase adds a separate RNA fragment to the 3' terminus of the original RNA template [5].

Historically, experiments with ssRNA coliphage mutants failed to provide evidence for recombination and the investigators concluded that RNA phages would not undergo recombination [8]. A potential flaw in the conclusion may have been that the study occurred at the time when FRNA phages were thought to possess only three genes, not four. In all likelihood, laboratory-applied selective pressure failed to detect or generate a specific recombinant. This failure may not necessarily reflect the lack of recombination or responsible mechanisms that could occur under actual environmental conditions encountered by ssRNA coliphages.

The first indication of RNA recombination in a male-specific FRNA phage was the report of small, non homologous, recombinant RNA molecules produced from a purified template-free Qβ replicase molecule [9]. The investigators noted similar RNA molecules were present in *E. coli* cells infected with phage Qβ. Chetverin *et al*., [7] studied this phenomenon by observing the formation of novel sequences in RNA molecules which suggested that this recombination event occurred as a transesterification reaction catalyzed by a conformation acquired by Qβ replicase during RNA synthesis [5,7]. Nucleotide sequences of recombined RNA molecules non-homologous to the parent RNA were formed in the absence of DNA intermediates, demonstrating an RNA recombination mechanism in the presence of Qβ replicase [5]. Therefore, it was plausible to have recombination in environmental ssRNA male-specific coliphage (*Leviviridae*) isolates. 

In the present study, two JS strains, DL52 and DL54, were isolated during an environmental genotyping study of *Leviviridae* FRNA phages [10,11]. As in the Vinjé study [4], strains DL52 and DL54 were isolated from separate coastal waters. These phages were placed into the putative JS subgroup using the genotyping methods of Vinjé *et al*. [4]. The objective of this study was to determine whether the existence of a novel JS-like subgroup representing a third *Levivirus* cluster as proposed by Vinjé *et al*., [4] could be verified. The approach taken here was to compare sequences from the JS strains to nucleotide and amino acid sequence data from entire genomes of 10 levivirus genogroup I strains and 5 levivirus genogroup II strains [3]. Analysis of the novel JS strains provided evidence to determine whether these *Levivirus* strains clustered to genogroup I, II, a combination of groups I and II or a unique genogroup. To further understand the phylogeny of these JS strains, complete genomic sequencing, amino acid composition, phylogenetic, bioinformatic and statistical analyses were performed.

## 2. Results

### 2.1. Sequence Analyses and Open Reading Frames

Preliminary analysis of nucleotide sequences from a replicase 189 bp amplicon placed the two novel strains, DL52 and DL54, into a “JS-like” subgroup [4]. Reverse-line blot hybridization failed to genotype the two strains into genogroups I or II [4]. 

A total of 17 strains (Table 1) were used to examine the relationships among nucleotides and amino acids in the *Levivirus* genus. The first 9 strains in genogroup I, Table 1, *i.e.*, MS2, ST4, DL1, DL2, DL13, DL16, R17, M12 and J20, were referred to as “MS2-like.”

Genogroup I MS2-like strains Open Reading Frame (ORF) start and stop codons were located at identical or very similar nucleotide positions as previously reported for strain MS2. The JS strains also had identical ORF start and stop codon positions as the MS-2 like strains (Table 2).

Nucleotide pairwise comparisons of full-length genomes were made between all strains within the *Levivirus* genome, including strains within genogroups I, JS and genogroups II. Within the nine strains of MS2-like genogroup I, full-length nucleotide sequence similarity was 91–99% [3] whereas the two JS strains, DL52 and DL54, shared 96.73% sequence similarity to each other. In comparison, the JS nucleotide sequences were more similar to MS2-like genogroup I (80–85%) than to the genogroup I strain fr (69%) or to genogroup II strains (52–54%) (Table 3 a).

**Table 1:** Male-specific ssRNA coliphages (FRNA), family *Leviviridae*, genus *Levivirus*, strain origins and identifications.

Strain	Genogroup	Source	Origin	Accession number
MS2	I	sewage	Berkeley, CA	**NC_001417**
M12	I	sewage	Germany	**AF195778**
DL1	I	river water	Tijuana River, CA	**EF107159**
DL2	I	bay water	Delaware Bay, DE	N/A
DL13	I	oyster	Whiskey Creek, NC	N/A
DL16	I	bay water	Great Bay, NH	**EF108464**
J20	I	chicken litter	South Carolina	**EF204939**
ST4	I	unknown	unknown	**EF204940**
R17	I	sewage	Philadelphia, PA	**EF108465**
fr	I	dung hill	Heidelberg, Germany	**X15031**
DL52	I-JS	bay water	Rachel Carson Reserve, NC	**JQ966307**
DL54	I-JS	bay water	Narragansett Bay, RI	**JQ966308**
GA	II	sewage	Ookayama, Japan	**NC_001426**
KU1	II	sewage	Kuwait	**AF227250**
DL10	II	mussel	Tijuana River, CA	**FJ483837**
DL20	II	clam	Narragansett Bay, RI	**FJ483839**
T72	II	bird	Talbert Marsh sandflats, CA	**FJ483838**

**Table 2:** Open Reading Frame positions and genome lengths of FRNA coliphage (family *Leviviridae*, genus *Levivirus*). Nucleotide positions are based on alignment. Number of amino acids for each gene is in parentheses [3].

Open Reading Frame Locations (amino acids)						
Strain	Group	Full length	ORF1	ORF2	ORF3	ORF4
MS2 ^a^	I	3569	130-1311(393)	1335-1727(130)	1678-1905(75)	1761-3398(545)
M12 ^a,b^	I	3340^b^	130-1311(393)	1335-1727(130)	1678-1905(75)	ND
DL1	I	3570	130-1311(393)	1335-1727(130)	1678-1905(75)	1761-3398(545)
DL2	I	3491^c^	130-1311(393)	1335-1727(130)	1678-1905(75)	1761-3398(545)
DL13	I	3491^c^	130-1311(393)	1335-1727(130)	1678-1905(75)	1761-3398(545)
DL16	I	3569	130-1311(393)	1335-1727(130)	1678-1905(75)	1761-3398(545)
J20	I	3569	130-1311(393)	1335-1727(130)	1678-1905(75)	1761-3398(545)
ST4	I	3569	130-1311(393)	1335-1727(130)	1678-1905(75)	1761-3398(545)
R17	I	3569	130-1311(393)	1335-1727(130)	1678-1905(75)	1761-3398(545)
fr ^a^	I	3575	129-1310(393)	1336-1728(130)	1691-1906(71)	1762-3399(545)
DL52	JS	3525	130-1311(393)	1335-1727(130)	1678-1905(75)	1761-3398 ^d^ (545)
DL54	JS	3398 ^c^	130-1311(393)	1335-1727(130)	1678-1905(75)	1761-3398 ^d^ (545)

^a^ previously published in GenBank; ^b^ strain M12 was not fully sequenced in the GenBank submission; ^c^ nearly full-length genome; ^d^ contains numerous deletions and insertions in ORF4; ND = not determined.

**Table 3:** (**a**) Pairwise nucleotide full-length genome percent similarity. (i) *Levivirus* JS strains DL52 and DL54 compared to genogroup I; (ii) *Levivirus* JS strains DL52 and DL54 compared to genogroup II. Pairwise alignments were performed in BioEdit with DAYHOFF similarity parameters.

(i) Genogroup I and JS strains		
Strain	DL52	DL54
DL52	100	
DL54	96.73	100
DL1	81.48	81.87
DL16	85.41	84.72
ST4	80.30	80.11
R17	80.55	80.53
J20	82.00	82.01
MS2	80.12	80.01
fr	69.18	69.06
**(ii) Genogroup II and JS strains**		
**Strain**	**DL52**	**DL54**
DL52	100	
DL54	96.73	100
T72	53.96	53.53
DL10	54.07	53.89
DL20	52.87	52.65
GA	52.44	52.29
KU1	52.94	52.66

Despite their sequence similarities, genome lengths for JS strains (3525 nt) were shorter than all genogroup I strains (3569–3575 nt) (Table 2) but longer than genogroup II (3458–3486 nt) [3]. Numerous deletions in the 3' untranslated region and a portion of ORF4 (replicase) in JS strains accounted for the decreased genome length (data not shown) but did not alter the ORF positions when the genogroup I strains were aligned (Table 2).

**Figure 1:**
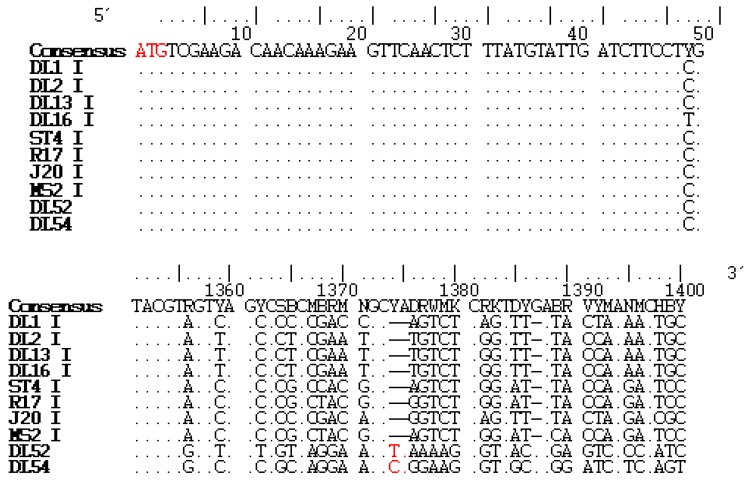
Replicase recombinant region in two JS strains when compared to genogroup I strains (family *Leviviridae*). Alignment (BioEdit v7.0.1) of the replicase nucleotide sequences from *Levivirus* genogroup I strains DL1, DL2, DL13, DL16, ST4, R17, J20, MS2 with JS strains DL52 and DL54. For clarity, only a portion of the alignment is shown. Alignment of each genogroup is depicted in discontinuous blocks. The numbers along the top are the nucleotide positions within the replicase gene with the start position of ORF4 assigned as nucleotide 1. Genome sequences read 5'-3' direction. Dots indicate identity with the consensus sequence. Degenerate bases are noted in the standard IUB codes. The replicase start codon and two nucleotide insertions are highlighted in red. Dashes denote a nucleotide sequence deletion from the consensus sequence.

Analysis of the replicase gene revealed a 2 nt insertion at the 1374 nucleotide region when counting ORF4 start site as nucleotide 1 (Figure 1). This insertion occurred upstream from the ORF4 stop codon. Beginning approximately 40 nt downstream from the replicase ORF4 stop codon and continuing to the 3' termini, 53 nt deletions were present in the JS strains when aligned to MS2-like genomes. Nucleotide alignment of the replicase and nontranslated regions (NTR) revealed numerous nt deletions in the JS strains when compared to genogroup I strains accounting for the change in amino acid composition. However, JS strains shared the 3' terminal “signature”, ACCACCCA, present in *Levivirus* genogroups I and II [3]. 

### 2.2. Amino Acid Analysis

Initially, nucleotide pairwise analyses of full-length genomes were made comparing all strains within the *Levivirus* genome, including genogroups I, JS and II; an 80–85% nucleotide similarity between JS strains and the MS2-like strains was observed (Table 3 a). In comparison, the amino acid sequences of the maturation, capsid and lysis proteins of the JS strains were very similar to those of the MS2-like genogroup I strains, sharing 97–100%, 98–100% and 95–100% sequence similarities, respectively (Table 3 b). Genogroup I strain fr, when compared to MS2-like and JS genogroup I strains, only shared an amino acid similarity to the maturation, capsid and lysis proteins ranging from 75.73–91.85% (Table 3 b). In contrast, the replicase protein sequences of the JS strains were quite dissimilar to the replicase protein sequences of the MS2-like genogroup I strains, displaying a similarity range of 79–85% (Table 3 c). However, a similarity of 97–99% was observed among the highly conserved replicase genes for the MS2-like strains. Strain fr shared a 79% replicase similarity to JS strains and approximately 88–89% similarity to MS2-like strains. Genogroup II replicase was approximately 52–53% similar to JS strains, 50–53% to MS2-like and fr strains and 92–98% similar to other genogroup II strains (Table 3 c). 

**Table 3:** (**b**) Percent similarity in amino acid sequences between *Levivirus* JS strains and genogroup I maturation, capsid and lysis proteins. Amino acid pairwise computations were performed in Bionumerics.

Maturation Protein
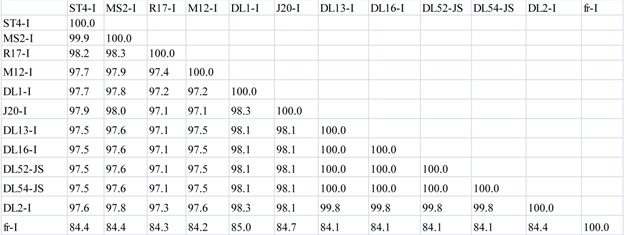
**Capsid Protein**
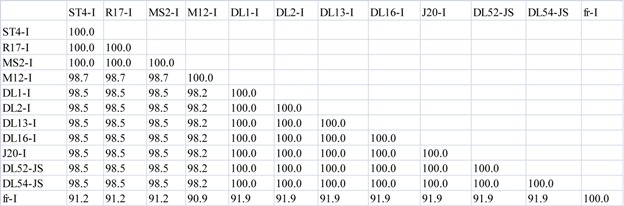
**Lysis Protein**
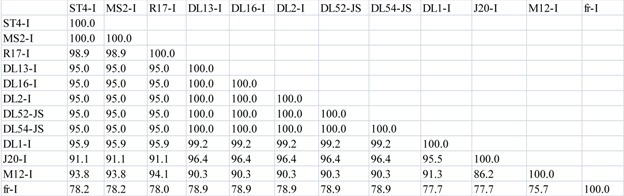

**Table 3:** (**c**) Amino acid percent similarity comparisons between *Levivirus* JS strains, DL52 and DL54, to *Levivirus* genogroup I and genogroup II RNA-dependent RNA polymerase (replicase) protein. Amino acid pairwise computations were performed in Bionumerics.

Replicase Protein
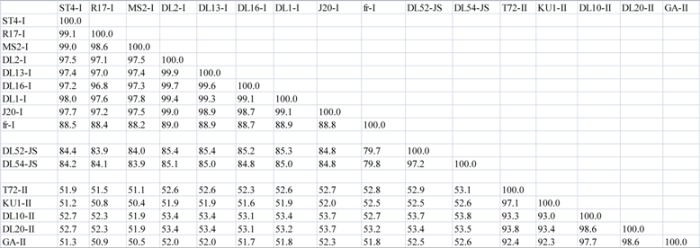

All genogroup I strains, including fr, and the two JS strains had a replicase protein length of 545 amino acids (Table 2) [3]. However, JS replicase differed from genogroup I replicase as it had one amino acid insertion at replicase position 467 and one amino acid deletion at the 3' termini of the stop codon, but maintained a total of 545 amino acids (data not shown). Identical to genogroup I strains, the replicase catalytic domain in the JS strains occurred between amino acid positions 243–373, thereby adding confidence to placing the grouping of JS into genogroup I [3]. Beginning at amino acid number 455 within the replicase gene, JS strains were unique in amino acid composition and diverged from the MS2-like strains. 

### 2.3. Pfam and Protein Sequence Motifs

Individual proteins from JS strains DL52 and DL54 were grouped to protein families by Pfam analysis. The maturation protein generated “phage_mat-A” domain including all *Leviviridae* strains plus three additional bacteriophage, PRR1, PP7 and AP205. The capsid protein resulted in a “Levi-coat” domain including all *Leviviridae* strains plus bacteriophage PRR1. The lysis protein only generated results in a PfamB search matching the genus *Levivirus* strains from both genogroups I and II including KU1, JP34, M12, FP501, MS2, JP500, fr, TH1, SD, GA, BO1, TL2 and ZR. Replicase protein matched “RNA_replicase_B” domain within the *Leviviridae* family plus the additional bacteriophages PRR1, PP7 and AP205.

Common protein motifs such as casein kinase II phosphorylation, cAMP and cGMP-dependent protein kinase phosphorylation and protein kinase C phosphorylation occurred in DL52 and DL54 when compared to the *Levivirus* strains [3]. Interestingly, every amino acid motif position in all four genes was identical among these two JS strains. 

### 2.4. Phylogenetic and Recombination Analyses

**Figure 2:**
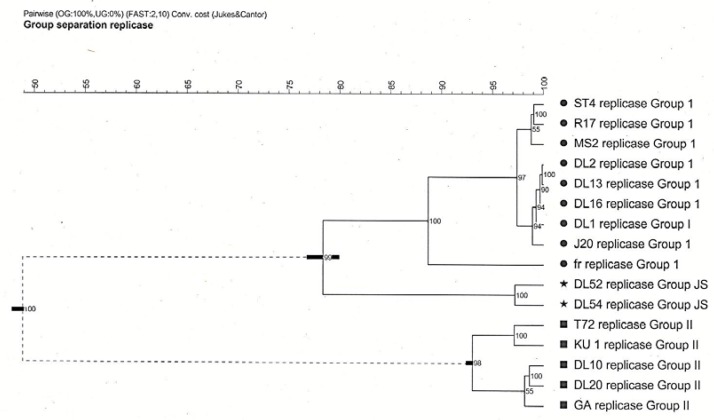
Cophenetic cluster analysis of *Levivirus* (family *Leviviridae*) genogroups I and II strains generated from pairwise similarities of the replicase amino acid sequences. Horizontal bars at three of the branches show the standard deviations of the average similarities of the clusters. Numbers at each branch are the cophenetic correlations which represent the faithfulness of the clusters. Two relevant clusters, as determined by the cluster Cutoff method, are grouped as dictated by the dashed lines. Analysis performed in Bionumerics.

Cophenetic correlations showed the genogroup I strains, the JS subgroup strains, and the genogroup II strains all formed faithful clusters with correlations of 100, 90 and 98, respectively. The cluster cutoff method, however, showed only two relevant clusters being the genogroup I strains, which included fr and JS, and genogroup II strains (Figure 2). 

When referring to nucleotide or amino acid positions within the replicase gene, the numbering is in reference to the start codon as being position 1. In all analysis programs, the nucleotide or amino acid sequences were aligned to other strains and were therefore approximate positions on the replicase gene. 

All recombination programs used, SimPlot, RAT, RDP3 and Recco, statistically predicted recombination in both JS strains, DL52 and DL54, when compared to genogroup I MS2-like strains. No recombination, however, was detected when DL52 and DL54 were compared to genogroup I strain fr and all genogroup II strains. 

**Figure 3:**
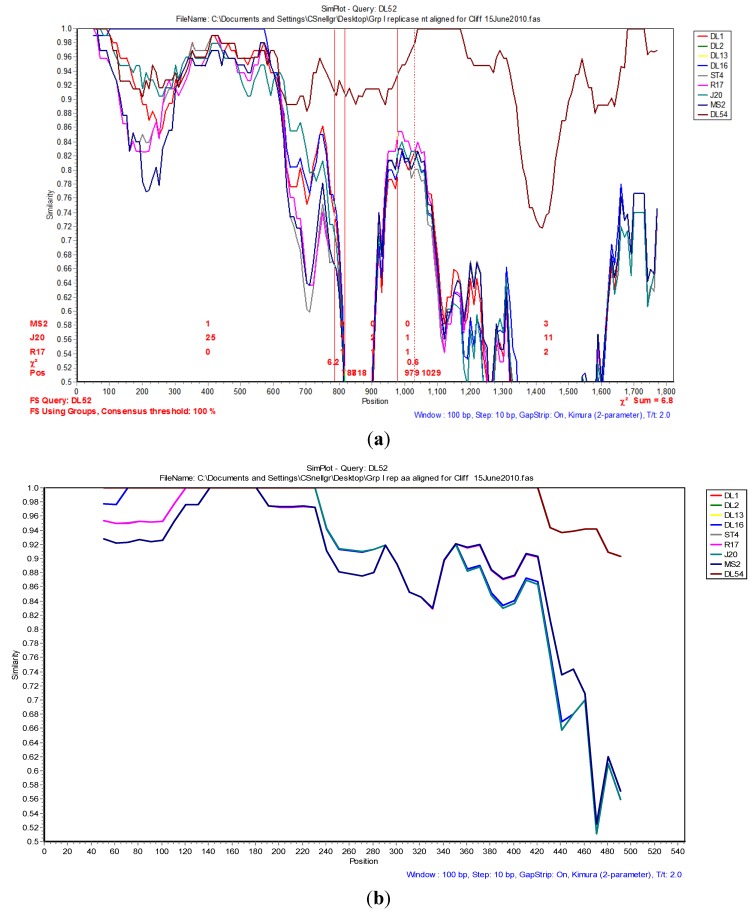
(**a**) The Simplot and bootscan analyses of the replicase nucleotides from JS strain DL52 queried to DL54, DL1, DL3, DL13, DL16, ST4, R17, J20 and MS2. The breakpoints are shown by the vertical red lines. The first recombination breakpoint occurred in the replicase gene in strain DL52 at nucleotide positions 787–818 where the χ^2^ changed from 0.8 to 6.3 (sum χ^2^ of 7.1). The second breakpoint occurred at nucleotide positions 979–1029 where the χ^2^ changed from 0.6 to 7.0 (sum χ^2^ of 7.6); (**b**) The Simplot and bootscan analyses of the replicase amino acids from JS strain DL52 queried to DL54, DL1, DL3, DL13, DL16, ST4, R17, J20 and MS2.

The Simplot and bootscan analyses of the replicase nucleotides from JS strains DL52 compared to *Levivirus* genogroup I strains DL54, DL1, DL3, DL13, DL16, ST4, R17, J20 and MS2 is shown in Figure 3A. Since the replicase nucleotide sequences in strain DL54 were 97% similar to strain DL52, DL52 was chosen as the query. The SimPlot analysis revealed the first recombination breakpoint occurred in the replicase from strain DL52 at nt positions 787–818 (approximate amino acid 262–273) where the χ^2^ changes from 0.8 to 6.3 (sum χ^2^ of 7.1). The second breakpoint occurred at nt positions 979–1029 (approximate amino acid 326–343) where the χ^2^ changes from 0.6 to 7.0 (sum χ^2^ of 7.6). However, Simplot amino acid analysis (Figure 3 b) with strain DL52 showed a divergence at approximate amino acid position 460 region which is in agreement with the manual alignment (Figure 1).

**Figure 4:**
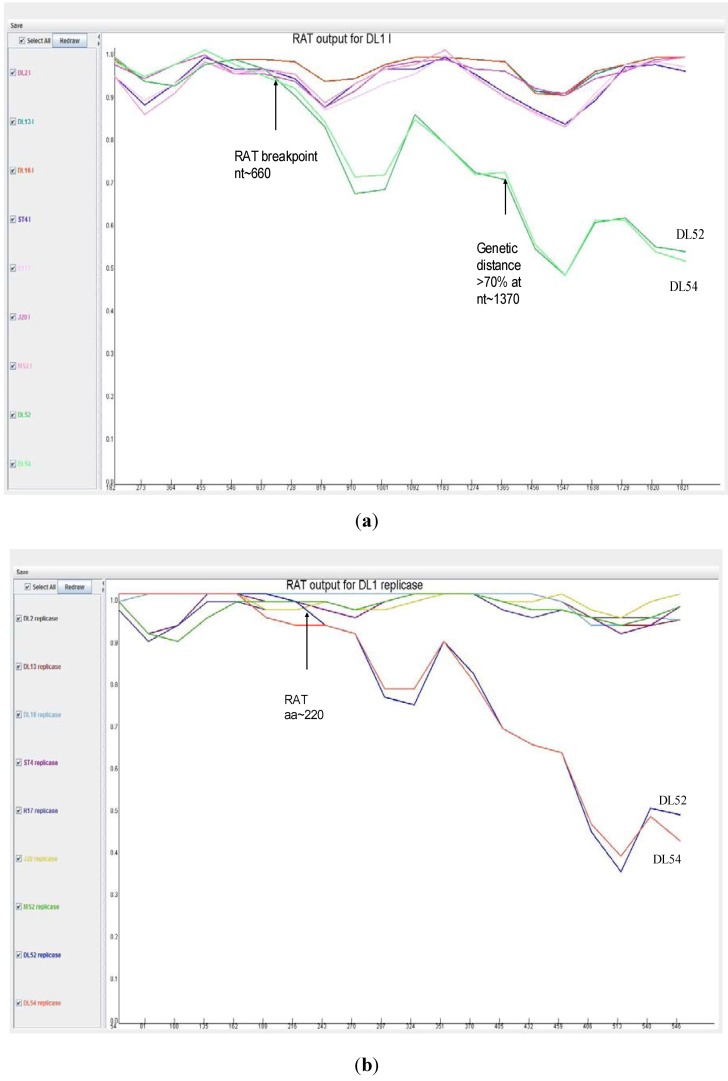
Recombination analysis of the replicase nucleotide sequences from *Leviviridae* genogroup I strains DL13, DL16, ST4, R17, J20, MS2 and JS strains DL54, DL52 queried to DL1. Recombination Analysis Tool (RAT) was used to generate graphics with a window of 182 nt and step increments of 92 nt. The Y-axis represents the genetic distance and the X-axis is the sequence location along the genome. (**a**) The JS strains, depicted in green, diverged from the other genogroup I strains at approximate nucleotide (nt) position 660; (**b**) Recombination analysis of the replicase amino acid sequences from *Leviviridae* genogroup I strains DL13, DL16, ST4, R17, J20, MS2 and JS strains DL54, DL52 queried to DL1. Recombination Analysis Tool (RAT) was used to generate graphics with a window of 54 aa and step increments of 27 aa. The JS strains, DL52 and DL54, diverged from the other genogroup I strains at approximate amino acid 220 within the replicase gene.

When analyzed with RAT, the nucleotide breakpoint (crossover) positions occurred at approximately nt 660 (Figure 4 a) or amino acid 220 (Figure 4 b) within the replicase gene. This crossover occurred when the two recombinant strains, DL52 and DL54, crossed the lines of the other MS-2 like strains and diverged by increasing genetic distances. 

**Figure 5:**
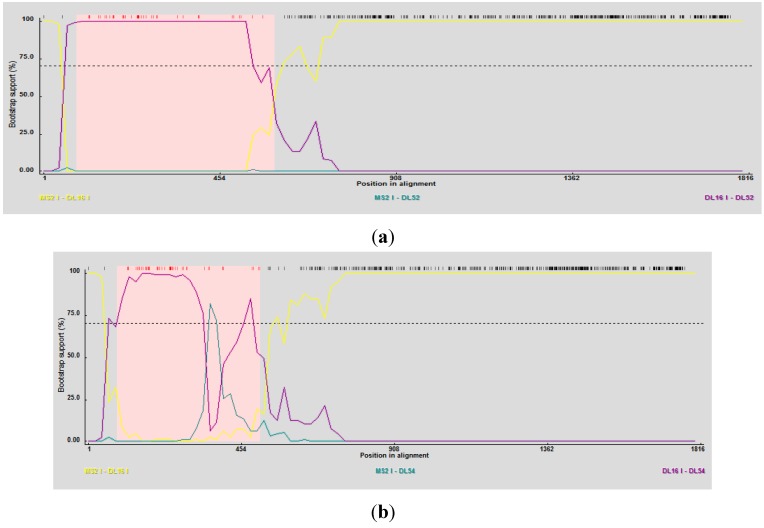
(**a**) RDP3 analyses prediction of DL52 as a recombinant strain. Recombination area within the replicase gene is shown in pink beginning at nucleotide 84 and crossing over at 592, upstream from the catalytic domain. DL52 was queried to all *Levivirus* (family *Leviviridae*) genogroup I FRNA *Levivirus* strains. RDP3 suggested DL16 as the minor parental strain; (**b**) RDP3 analyses predicted DL54 as a recombinant strain. Recombination area within the replicase gene is shown in pink beginning at nucleotide 84 and crossing over at 401, upstream from the catalytic domain. RDP3 suggested DL16 as the minor parental strain.

RDP3 predicted DL52 and DL54 as the recombinant strains using several detection methods and analysis algorithms (Table 4) and suggested DL16 as a minor parent strain. Breakpoint nucleotides for strains DL52 and DL54 (when aligned to genogroup I strains) occurred between nt 84–592 and 84–401, respectively (Figure 5 a, b), corresponding to the approximate amino acid breakpoint positions of 133–197 within the replicase gene.

**Table 4:** Prediction of DL52 and DL54 as recombinant strains by analysis of *Levivirus* (family *Leviviridae*) genogroup I using Recombination Detection Program (RDP3).

Confirmation Table of Recombination Events		
Methods	Events	Average p-value
RDP	2	2.199 × 10^−15^
GENECONV	1	3.031 × 10^−27^
Bootscan	2	7.867 × 10^−19^
MaxChi	2	1.445 × 10^−10^
Chimaera	2	3.536 × 10^−11^
SiScan	1	1.168 × 10^−13^
3Seq	1	4.486 × 10^−8^

Manual alignment in BioEdit of the replicase nucleotides, counting the ATG start codon of the replicase gene as nt 1, showed an insertion of the nucleotides YA beginning at position 1374 (Figure 1) whereas the amino acid composition of the JS strains diverged from the other genogroup I strains slightly upstream from this insertion at amino acid position 455 (nucleotide 1366). Alignment also revealed numerous nt deletions as discussed in the “Sequence analyses and ORF” section.

**Figure 6:**
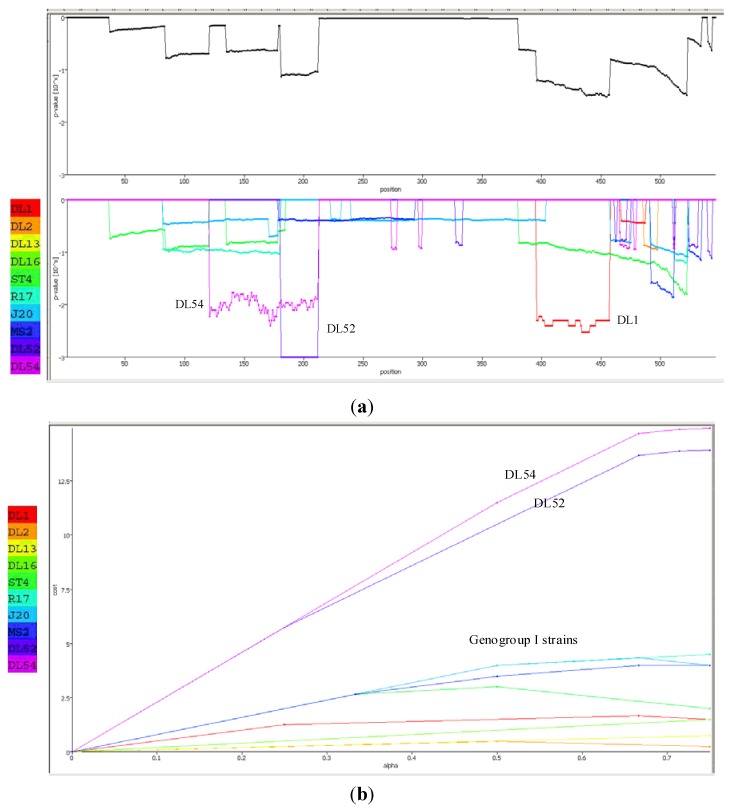
(**a**) Recco analysis of the RNA-dependent RNA polymerase (replicase) amino acid sequences in *Levivirus* genogroup I male-specific coliphages (FRNA). Recombination events are displayed by downward peaks in the graphics dataset. The upper graph represents the p-value for recombination at each position along the replicase gene. The lower graph is the breakpoint p-values for the entire set of *Levivirus* genogroup I and JS strains DL52 and DL54; (**b**) Recco parametric cost curve analysis of the RNA-dependent RNA polymerase (replicase) amino acid sequences for each FRNA strain in *Levivirus* genogroup I and JS strains DL52 and DL54. The y-axis corresponds to the cost curve and the x-axis represents α (0–1).

The Recco p-value inspector predicted strain DL52 had recombined with strain DL1 (Figure 6a). In DL52, the recombinant region spanned from amino acids 181–212 whereas the DL1 region spanned from 396–457 with resulting sequence p-values of 0.000999 and 0.004995, respectively. Recco parametric cost curves predicted the highest preference for recombination in strains DL52 and DL54 (cost of 12.5–13) whereas the remaining genogroup I strains did not show a preference for recombination (cost of 0–3) (Figure 6 b).

RAT, RDP3 and Recco all predicted recombination breakpoints ranging from amino acid positions 181–252 whereas Simplot agreed most closely with the manual alignment of 460 and 455, respectively. Also in agreement with the manual alignment was the crossover region between DL52 and DL1 occurring in the approximate amino acid region of 396–457 (Figure 6 a). The predicted breakpoint regions occurred either upstream or downstream from the highly conserved catalytic domain amino acid positions 243–373 in *Levivirus* genogroup I [3].

## 3. Discussion

Reported here are whole genome sequence data and bioinformatic analyses supporting the hypothesis that two novel FRNA isolates, DL52 and DL54, were the result of natural recombination. Initially classified as a JS subgroup of genogroup I within the *Levivirus* genus (family *Leviviridae*), these strains were isolated from seawater approximately 1130 km apart in the Rachel Carson Reserve, Beaufort, NC, and Narragansett Bay, RI. Findings that JS strains were highly similar to three out of four genes (maturation, capsid and lysis) in genogroup I MS2-like strains, shared the catalytic site location in the RNA-dependent RNA polymerase (replicase) gene, had an identical 3’ signature [3] and then greatly diverged along a stretch of the replicase gene all supported the occurrence of a recombination event. 

 In this study, two JS strains shared >95% amino acid identity in three (maturation, capsid and lysis) *Levivirus* genogroup I MS2-like genes but only an 84–85% amino acid identity to the otherwise, highly conserved replicase protein. In comparison, genogroup I strain fr was uniformly different from all other genogroup I strains in all four proteins [3]. Cophenetic correlations and bootstrap analysis strengthen the possibility that JS strains were recombinants as the JS strains were only a subgroup of genogroup I and not a novel genogroup. Throughout the *Leviviridae* family, subgroups emerge within genogroups, however, subgroup strains differ in all four genes from the parent genogroup [3]. It is therefore plausible to propose natural recombination in these two novel JS-like FRNA coliphages as data presented here suggested a specific genetic rearrangement or recombination event in the replicase gene.

Interestingly, different *Leviviridae* subgrouped strains originating from across the globe display high amino acid similarity among subgrouped strains [3]. Recombination may explain why *Leviviridae* strains circulate as discrete subgroups independent of geographical location. Although the two unique JS-like strains were isolated from NC and RI, they shared 96.73% nucleotide similarity across the entire genome. Thus, either a single, natural recombination event occurred as a *de novo* mutation in each strain or identical, natural recombinations along a hot spot in these genomes formed these strains. In either case discovering that geographically-separated JS-like strains acquired the same recombination event is intriguing. 

Largely responsible for the diversity of RNA viruses [12] RNA-RNA recombination was observed in several positive-sense, ssRNA human and animal viral taxa including caliciviruses, coronaviruses, hepatitis, dengue, enteroviruses and astroviruses [13,14,15,16,17,18,19,20,21]. For example, genetic exchange in ssRNA viruses was first demonstrated in polioviruses [22,23]. 

Recombination events frequently alter the RNA-dependent RNA polymerase region. Human Noroviruses, a positive sense ssRNA virus with a genome length of 7400–8300 nt, are considered to belong to a prototype strain if they share approximately 85% overall nucleotide sequence identity and a high amino acid sequence identity (>95%) in the polymerase gene [20]. A naturally occurring human Norovirus strain shared 95% amino acid sequence identity with the capsid sequences from a Mexico cluster and 95% amino acid identity to the polymerase in a Lordsdale virus cluster. Sequences from the natural strain were obtained from one viral isolate. The combination of sequences in the one strain being complementary to two distinct human Norovirus clusters led to the proposition that this strain was a naturally occurring recombinant [20].

Genetic recombination is known to occur in certain Enteroviruses, a positive ssRNA virus having an approximate 7500 nt genome. Poliovirus recombination occurs in vaccine-derived strains [24] in the human population as a single infected individual excretes a high proportion of recombinants [18]. To determine if other enteroviruses undergo natural recombination, isolates of echoviruses were collected from a meningitis outbreak. Nucleotide sequences were clustered based on a capsid protein (VP1) and RNA-dependent RNA polymerase (3D). Dendrogram relatedness of the echovirus strains grouped the VPI sequences to the prototype strains. However, the RNA polymerase sequences did not cluster to the prototype strains, suggesting genetic recombination among the outbreak strains [18].

Human astroviruses are positive sense, ssRNA with a genome length of approximately 6,800 nucleotides [19] and a polyadenylated 3' tail [21]. Two sets of strains were investigated for recombination; one set was identified from a child care center in Houston, TX, and the two other strains were found in stool samples from two children in Mexico City. The pool of strains shared >97% nucleotide sequence similarity in two out of three genomic regions. The novel strain clustered to one group based on the capsid region. When the RNA-dependent RNA polymerase was analyzed, the novel strain clustered to a separate human astrovirus group. The strains were identified as naturally occurring recombinants on the evidence of high sequence similarity to a few genes of one prototype and similarity to different genes in a second prototype. A total of 64 additional human astroviruses lacked these novel traits [19].

An enteric turkey astrovirus is a non-enveloped, positive sense ssRNA virus with a polyadenylated 3' tailed genome of approximately 7 kb. The most conserved gene in the avian and mammalian astrovirus is the RNA-dependent RNA polymerase or replicase. Genetic analysis of capsid and polymerase sequences from twenty-three turkey astrovirus strains resulted in 8 clusters for the capsid gene and two phylogenetic clusters for the RNA polymerase gene. Computer-generated analyses identified polymerase gene recombination in strains of turkey astrovirus [14].

In this study, four different recombination detection programs along with manual alignment predicted strains DL52 and DL54 as recombinants although the exact amino acid and/or nucleotide breakpoint varied somewhat along the RNA-dependent RNA polymerase gene. As expected the breakpoints did not occur within the catalytic-site domain. The sliding window approach as used with many recombination programs is based on an arbitrarily chosen window length, thus affecting the sensitivity and accuracy when pinpointing the precise breakpoint [25]. Overall, the use of a variety of recombination algorithms provided a stronger, more rigorous scientific case. When comparing the *Levivirus* strains, manual alignment provided a more accurate picture of where the recombination event occurred along the genome but it did not provide a statistical analysis. Therefore, statistical analysis in combination with manual alignment resulted in a more confident assessment of recombination. Evidence for recombination among positive ssRNA viruses exists within the RNA-dependent RNA polymerase (replicase) gene for numerous viruses as described here and supports the data that natural recombination can occur within the *Leviviridae* family. 

## 4. Experimental Section

### 4.1. FRNA Coliphage Strains and RNA Extraction

FRNA phage strains CICEET 29 and CICEET 24 were isolated and placed into the putative JS subgroup [10,11] using the genotyping methods of Vinjé *et al*., [4]. CICEET 29, renamed DL52, was isolated from estuarine waters in Rachel Carson W Reserve (Beaufort), NC, and CICEET 24, renamed DL54, and was isolated from Narragansett Bay, RI (Table 1).

Each strain was plaque purified and further enriched using *Escherichia coli* HS(pFamp)R as host [4]. Approximately 1–2 mL aliquots of the purified viral supernatant were frozen at &75 °C. Coliphage RNA was extracted from purified virus as described [26] using a QIAamp viral RNA mini kit (Qiagen, Valencia, CA, USA). Purified RNA was stored frozen at &20 °C. 

### 4.2. Sequencing and Analysis

Full-length genome sequencing was performed by the “primer walking” approach as described [3]. Nucleotide and amino acid sequences from JS strains DL52 and DL54 were compared to nucleotide and amino acid sequences from 10 genogroup I strains (MS2, DL1, DL2, DL13, DL16, ST4, R17, J20, M12, fr) and 5 genogroup II strains (T72, DL10, DL20, GA, KUI) [3]. 

Nucleotide sequences from three individual clones were imported and aligned using BioEdit v7.0.1 [27] followed by Basic Local Alignment Search Tool (BLAST, National Center for Biotechnology Information) analyses for sequence and phylogenetic confirmation. Completed sequences from all strains were aligned with full-length prototype strains (GenBank) using BioEdit ClustalW application. For each strain, the Open Reading Frames (ORFs) were mapped using BioEdit.

### 4.3. Amino Acid Analysis

Deduced amino acid sequences for each of the four genes were determined using a computer-generated DNA-to-protein translation tool, ExPASY (http://ca.expasy.org/). Prediction of protein sequence motifs were identified by PROSITE (http://ca.expasy.org/) and protein families and domains were modeled in Pfam (http://pfam.janelia.org).

### 4.4. Phylogenetic, Statistical and Recombination Analyses

Sequence data were analyzed using BioNumerics Software v.3.5 (Applied Maths, Saint-Martens-Latem, Belgium). Phylogenetic trees were built by global cluster analysis performed on multiple aligned sequences and clustered by unweighted pair group method using arithmetic averages (UPGMA). A bootstrap analysis, based on 10,000 substitutions, was used to measure cluster significance. The reliability of each cluster was expressed on a percentage basis [3]. 

Nucleotide percent similarity and dendrograms were constructed using BioNumerics Software v.3.5 (Applied Maths, Saint-Martens-Latem, Belgium). Phylogenetic trees were built by global cluster analysis performed on multiple aligned sequences and clustered by UPGMA using the Jukes and Cantor correction [28]. Cophenetic correlations and cluster Cutoff method were employed to measure faithfulness and relevancy of the clusters (Applied Maths, Saint-Martens-Latem, Belgium). Average similarities with standard deviations were calculated for the relevant clusters. 

Various approaches were used to examine recombination in *Levivirus* FRNA strains, using aligned nucleotides and aligned amino acids, as follows: (i) manual alignment using BioEdit, (ii) bootscan analysis in SimPlot v3.5.1 [29] (iii) Recombination Analysis Tool v1.0 [30], (iv) Recombination Detection Program v3.44 [31], and (v) Recombination Analysis Using Cost Optimization (Recco) v0.93 [32] FRNA strains used in analyses were genogroup I strains DL1, DL2, DL13, DL16, ST4, R17, J20, MS2, fr; JS strains DL52 and DL54; and genogroup II strains GA, KU1, DL10, DL20 and T72.

SimPlot analyses was determined with a sliding window of 100 bp wide and a step size between plots of 10 bp when comparing reference strains to the queried sequences. Recombination events by SimPlot bootscan analysis occurred when the χ^2^ value changes signifying a breakpoint position. 

Aligned replicase amino acids and/or nucleotide sequences were analyzed in Recombination Analysis Tool (RAT), Recombination Detection Program (RDP3) and Recco. RAT uses a distance-based method of recombination in both DNA and protein multiple alignments [30]. Unless stated otherwise, default settings were used with each program. The RAT default settings were window size of 10% of the sequence length and an increment size being half of the window size. Both settings of “auto search” and “test sequence search” were used with RAT. 

Recombination Detection Program v3 (RDP3) uses a number of recombination detection algorithms such as RDP, Bootscan, GENECONV, Maximum Chi Square, CHIMAERA, Sister Scanning (SISCAN) and 3SEQ [31]. The RDP3 program sorts the analyses from these various algorithms and statistical data to determine the unique recombination events. RDP3 used an alignment of all genogroup I and JS replicase nucleotide sequences and queried to DL16, MS2, DL52 and DL54. 

The Recco p-value inspector was set at 3 and the permutation was set at 1000. Recco uses an algorithm that locates putative recombination points based on cost minimization. Recco compares the cost of mutation relative to recombination as represented by α. 

### 4.5. Nucleotide Sequence Accession Numbers

The accession numbers for DL52 and DL54 are JQ966307 and JQ966308, respectively. 

## 5. Conclusions

The results of this study provide genetic evidence, bioinformatic and statistical analyses suggesting a natural recombination event in the formation of a genogroup I subgroup JS-like *levivirus,* represented by two strains, DL52 and DL54. There was high nucleotide and amino acid identity in three genes, the maturation, capsid and lysis genes (≥95%) but a lack of similarity in the replicase gene (84–85%) when JS strains were compared to genogroup I MS2-like strains. Four different recombination programs demonstrated one or two breakpoint regions in the replicase gene, signifying a recombination event. The recombination event occurred downstream of the replicase catalytic site thereby maintaining viral integrity and replication function. Thus, primers for oligonucleotide hybridization probes targeting the replicase beyond the catalytic site would not hybridize to JS strains. In contrast, molecular assays targeting the maturation, capsid or lysis sequences would presumptuously place JS strains as an MS-2 like genogroup. 

Phylogenetic tree analysis produced a cophenetic correlation which showed (i) ten genogroup I strains, including strain fr, (ii) the JS subgroup strains, and (iii) the genogroup II strains all formed faithful clusters with correlations of 100, 90 and 98, respectively. The cluster cutoff method, however, revealed only two relevant clusters, (i) genogroup I strains, which included fr and JS, and (ii) genogroup II strains. Therefore, the novel JS strains are not a unique *Levivirus* genogroup. The proposed classification of JS strains is genogroup I subgroup “JS-like”.

Although both JS strains were prepared for sequencing in the same laboratory, these strains were field-collected by different investigators and shipped to another location where they were plaque-purified and preliminarily classified. Therefore, the possibility that contamination resulted in false recombinants seems unlikely. Likewise, the possibility of cloning and/or PCR errors contributing to the nucleotide and amino acids changes would not have led to both JS strains being almost identical in the non recombinant regions as seven genogroup I strains (DL1, DL2, DL13, DL16, R17, J20 and ST4), three genogroup II strains (DL10, DL20, T72) and two JS strains (DL52 and DL54) were sequenced in this lab in no certain strain or fragment order using the same methods and sequencing company [3]. Finally, to the best of our knowledge, this is the first description of recombinant viruses from natural isolates in ssRNA *Leviviridae* bacteriophages.
